# *Primotrapa* gen. nov., an extinct transitional genus bridging the evolutionary gap between Lythraceae and Trapoideae, from the early Miocene of North China

**DOI:** 10.1186/s12862-020-01697-2

**Published:** 2020-11-12

**Authors:** Ya Li, Yi-Ming Cui, Carole T. Gee, Xiao-Qing Liang, Cheng-Sen Li

**Affiliations:** 1 State Key Laboratory of Systematic and Evolutionary Botany, Institute of Botany, Chinese Academy of Sciences, Beijing, 100093 China; 2 State Key Laboratory of Palaeobiology and Stratigraphy, Nanjing Institute of Geology and Palaeontology and Center for Excellence in Life and Paleoenvironment, Chinese Academy of Sciences, Nanjing, Jiangsu Province, 210008 China; 3 Division of Paleontology, Institute of Geosciences, University of Bonn, Nussallee 8, 53115 Bonn, Germany; 4 Huntington Botanical Gardens, 1151 Oxford Road, San Marino, California, 92208 USA; 5 School of Chemical, Biological and Environmental Sciences, Yuxi Normal University, Yuxi, 653100 Yunnan Province China

**Keywords:** Water caltrop, Freshwater macrophyte, Extinct aquatic plant, *Primotrapa*, *Trapa*, *Hemitrapa*, Lythraceae, Miocene, Hebei Province, China

## Abstract

**Background:**

Although *Trapa* is a well-defined genus of distinctive freshwater plants with accumulations of extensive morphological and embryological autapomorphies, its phylogenetic relationships have long been unclear. Formerly placed in the monotypic family Trapaceae, *Trapa* is now recognized as sister to *Sonneratia* within Lythraceae s.l., although both genera lack morphological synapomorphies. Thus, a split between the two taxa must have occurred in deep evolutionary time, which raises the possibility of finding transitional forms in the fossil record.

**Results:**

Here we describe a new genus and species, *Primotrapa weichangensis* Y. Li et C.-S. Li (Lythraceae s.l.: Trapoideae), based on three-dimensionally preserved floral cups, fruits, and seeds from the early Miocene of Weichang County, Hebei Province, China. *Primotrapa* is characterized by a shallow, saucer-shaped floral cup, four distally barbellate sepals, four intersepal appendages alternating with the sepals at the rim of cup, a superior to basally inferior ovary, a fusiform or ovoid, one-seeded fruit with a ribbed surface, and a long persistent peduncle. Two fossil species of *Hemitrapa* are proposed as new combinations of *Primotrapa*, namely *P. alpina* (T. Su et Z.-K. Zhou) Y. Li et C.-S. Li comb. nov. and *P. pomelii* (Boulay) Y. Li et C.-S. Li comb. nov. Our phylogenetic analysis based on fifteen flower and fruit characters supports the placement of *Primotrapa*, *Hemitrapa* and *Trapa* in a monophyletic clade, which comprise subfamily Trapoideae. The phylogenetic analysis places *Primotrapa* at the base of Trapoideae.

**Conclusions:**

In view of its superior ovary, which is a plesiomorphic character of Lythraceae s.l., the newly recognized genus *Primotrapa* and its three species likely represent transitional forms that bridge the evolutionary gap between the basal taxa of Lythraceae s.l., i.e. *Lythrum*, and the highly derived taxon *Trapa*.

## Backgound

**Fig. 1:**
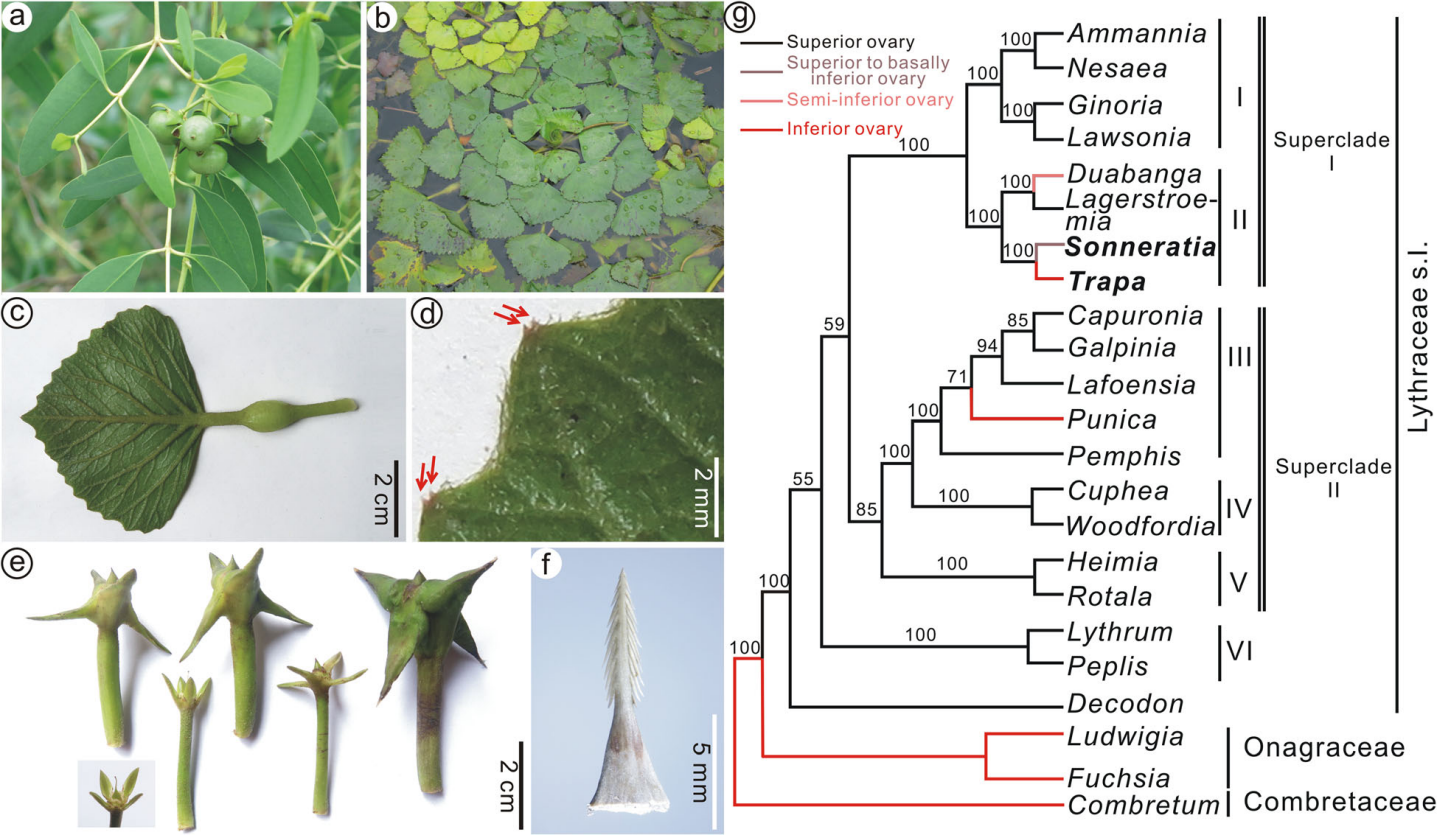
Extant *Sonneratia* and *Trapa* and the phylogenetic relationships of Lythraceae s.l. **a** *Sonneratia apetala* Buch.-Ham. from Qi’ao-Dan’gan Island Mangrove Nature Reserve, Zhuhai, Guangdong Province, China. **b** *Trapa natans* L. from Xuanwu Lake, Nanjing, Jiangsu Province, China. **c**, **d** A leaf of *T. natans* L. and an enlargement (d) showing the characteristic double apices of the teeth on the leaf margin (red arrows). **e** A series of fruits of *T. natans* showing different developmental stages. **f** A sepal of *T. natans* with the cortex removed, showing dital, recurved barbs. **g** The position of the ovary in Lythraceae s.l. mapped on the Bayesian 50% majority-rule consensus of 4000 trees from the combined molecular data of four gene regions [10, 11]

*Trapa* L., the water caltrop, is a genus of annual freshwater macrophytes, which grow in sluggish rivers, lakes, swamps, and ponds and are native to the tropical to warm temperate regions of Eurasia and Africa [1, 2]. *Trapa* has accumulated extensive autapomorphic features such as a unique embryo, leaf margins with characteristic double-mucronate tooth apices, distinctively horned fruits, distally barbellate sepals (horns) (Fig. 1b–f), and generally prolate-spheroidal crested pollen [3, 4]. This suite of striking characters led to the assignment of all living species in the genus to the monotypic family Trapaceae [1, 5, 6]. However, the relationship of the Trapaceae to other families has remained unclear. *Trapa* was once thought to be related to *Ludwigia* L. (Onagraceae) [7] or *Trapella* Oliv. (Pedaliaceae) [8], but any morphological similarities have been dismissed as homoplasy (non-homologous similarity) [6, 9]. A close relationship between *Trapa* and Lythraceae* sensu stricto *(s.str.) was also proposed, based on fossil evidence [9].

Molecular data unquestionably place *Trapa* and the other three distinct genera, namely *Duabanga* Buch.-Ham., *Punica* L., and *Sonneratia* L.f. in the family Lythraceae *sensu lato* (s.l.) (Fig. 1g) [10, 12–14]. From a morphological standpoint, Lythraceae s.l. lack morphological synapomorphies to define them, especially in regard to ovary position. It is a superior ovary in Lythraceae s.str., a superior to basally inferior ovary in *Sonneratia*, a semi-inferior ovary in *Duabanga*, and a nearly completely inferior ovary in *Punica* and *Trapa* [11]. Based on molecular phylogenetics, *Trapa* and *Sonneratia* are sister genera [10, 12–14], as unlikely as it may seem morphologically (Fig. 1a, b) [11]. Moreover, in regard to habit and habitat, *Sonneratia* is a tree growing in the brackish waters of mangrove and coastal environments, while annual *Trapa* is an aquatic plant floating in quiet bodies of freshwater. Hence, a link between *Trapa* and *Sonneratia* must exist in their common evolutionary history. Until now, the absence of this link in fossil record has greatly hampered our understanding of the evolutionary position of *Trapa* within Lythraceae s.l. and has led to a conflict between morphological and molecular systematics [10, 11].

While fossil remains of *Trapa* have been frequently reported from the Miocene swamp deposits of Europe and Asia [15–20], as well as from North America [21–26], unequivocal *Trapa* fossils first appear in the middle Miocene [2]. *Hemitrapa* Miki is an extinct genus of fossil fruits initially described by Miki [17] from the late Miocene of Akazu and Hatagoya, Japan, and is thought to be closely related to *Trapa* [9]. *Hemitrapa* fruits are fusiform and contain a semi-inferior ovary. They also occur frequently in other mid-latitude localities in the Miocene of Eurasia and North America [27] and disappear after the Pliocene [2, 27]. Based on comparative morphology, Miki hypothesized that living *Trapa* evolved from ancestral *Lythrum* L. through the extinct genus *Hemitrapa* [9]. However, Miki’s hypothesis can no longer be considered tenable, as *Trapa* and *Lythrum* share only a family level relationship [2]. While other studies also consider *Trapa* to be a descendant of *Hemitrapa* [4, 16], it is more likely that *Trapa* and *Hemitrapa* share a common ancestor [28]. It is worth pointing out that both *Trapa* and *Hemitrapa* are also morphologically very different from other taxa of Lythraceae s.l., including *Trapa*’s molecular genetic sister *Sonneratia*.

**Fig. 2:**
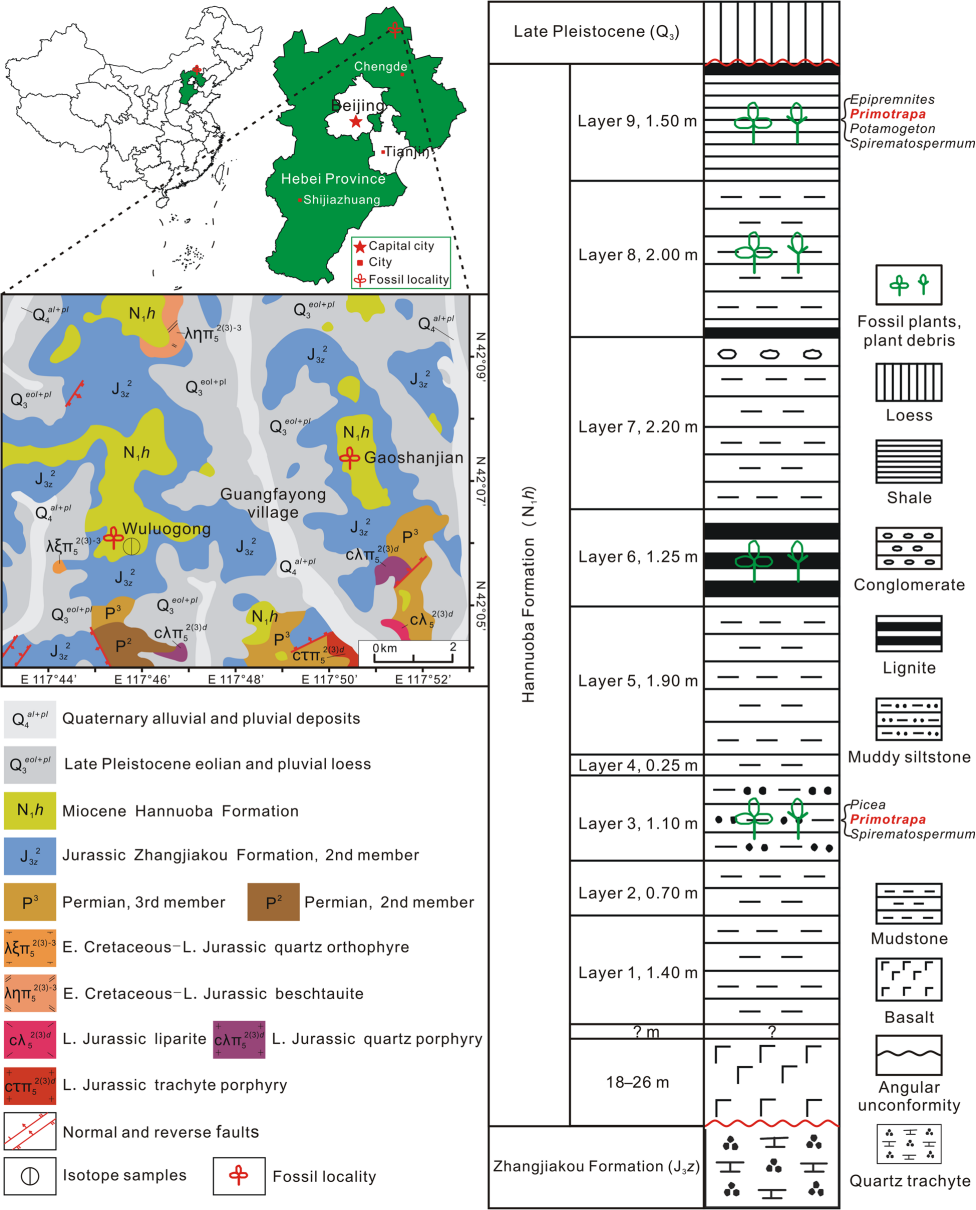
Geographic map, geologic map, and stratigraphic column of Gaoshanjian section in Weichang, Hebei Province, China, showing the locality and stratigraphic occurrences of *Primotrapa weichangensis*. The map of China was generated by the MapInfo Professional 8.5 SCP with the map data in Microsoft Office 2000. The geological map is modified from [29], and the stratigraphic column is modified from [30]

In the present study, we describe a new, extinct member of Lythraceae s.l., *Primotrapa weichangensis* Y. Li et C.-S. Li gen. et sp. nov., on the basis of three-dimensionally preserved floral cups, fruits and seeds from the early Miocene of Weichang County, Hebei Province, China (Fig. 2). A phylogenetic analysis shows that *Primotrapa*, *Hemitrapa*, and *Trapa* form a monophyletic clade, i.e. the subfamily Trapoideae. Phylogenetic analysis also reveals the transitional position of *Primotrapa* between *Sonneratia* and *Trapa* + *Hemitrapa* clade within Lythraceae s.l. The phytogeography of *Primotrapa*, its origin, and dispersal through geological time are also discussed.

## Results

### Systematic paleontology

Order: Myrtales Juss. ex Bercht. et J. Presl, 1820

Family: Lythraceae J. St.-Hil., 1805

Subfamily: Trapoideae Voigt, 1845

Genus: *Primotrapa* Y. Li et C.-S. Li gen. nov.

Generic diagnosis: Flowers perigynous or basally epigynous, bearing a short shallow, saucer-shaped floral cup, persistent on the fruit. Sepals 4, acicular, sparsely barbellate toward apex, inserted on floral axis in the same whorl, curved away from the floral axis, persistent on the fruit. Intersepal appendages 4, acicular, spiny, dichotomizing once, alternating with sepals at the rim of the cup, detaching from mature fruit. Ovary superior to basally inferior. Fruit fusiform, irregularly dehiscent, one-seeded, with an elongated apex, sparsely ribbed surface, and a long persistent peduncle.

*Etymology*: The genus name refers to its primitive *Trapa*-like fruits.

*Type species*: *Primotrapa weichangensis* Y. Li et C.-S. Li sp. nov.

*Specific diagnosis*: Floral cup perigynous with a superior ovary. Fruit fusiform or ovoid in shape, with an elongated and smooth (non-ribbed) apex. Barbs arise at a wide angle to the sepals. Fruit surface finely and sparsely ribbed.

**Fig. 3:**
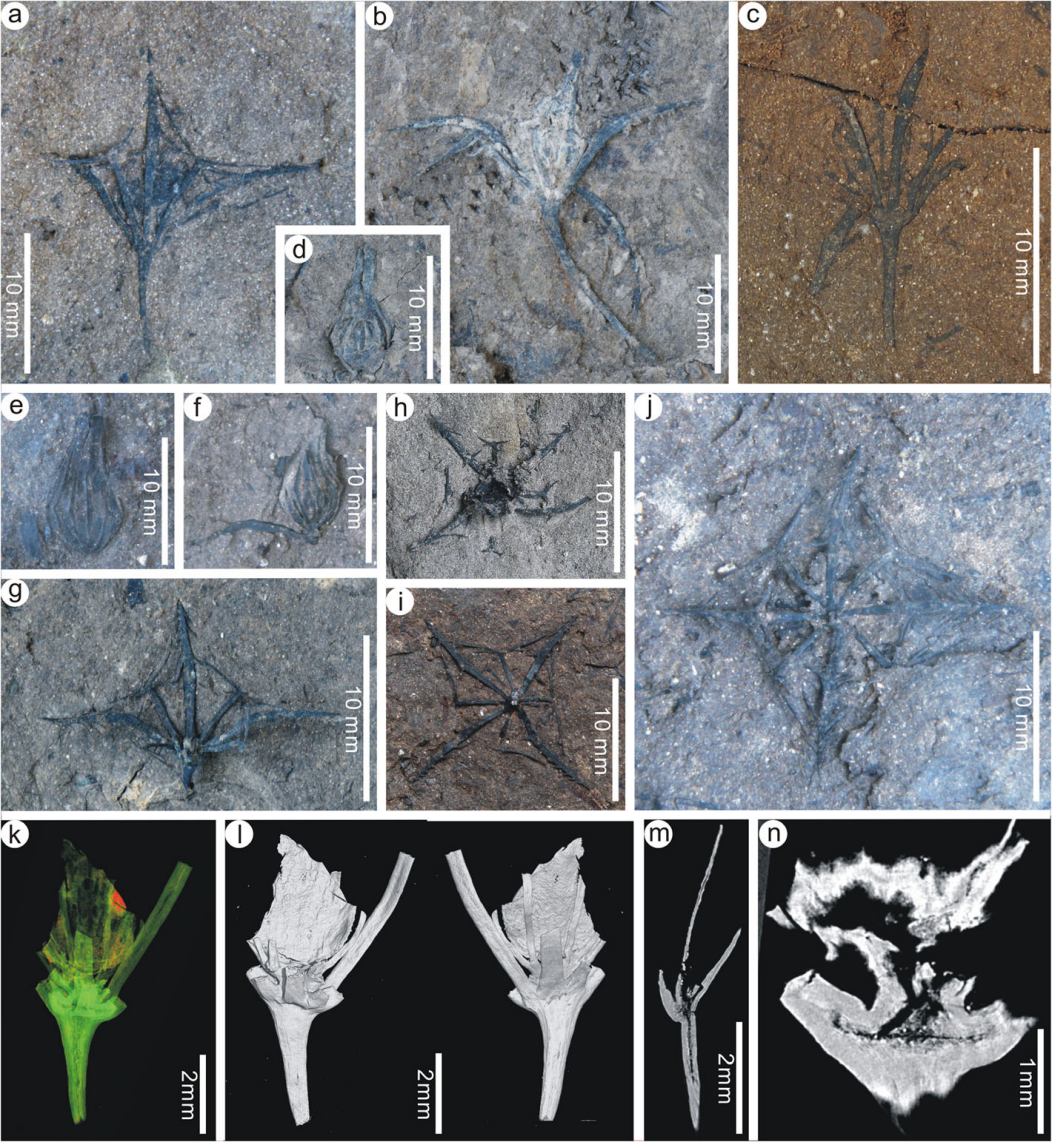
Floral cups and immature fruits of *Primotrapa weichangensis* from the Miocene of Weichang, China. **a** A laterally preserved, immature fruit showing the floral cup with three sepals and three intersepal appendages alternately arranged. PEPB70591 (Holotype). **b-c** Another two laterally preserved, immature fruits. PEPB70592–70593. **d-f** Abscised immature fruits showing the ribbed surface. PEPB70596–70598. **g-j** Laterally, distally, or basally preserved floral cups showing four sepals and four intersepal appendages alternately arranged at the rim of the cup. PEPB70601–70604. **k-n** Micro-CT-scanned, 3-D reconstruction of a floral cup (**k**) showing the front and back views of the floral cup (**l**), and the longitudinal sections (**m**, **n**). PEPB70605

*Holotype*: PEPB70591 (Fig. 3a).

**Fig. 4:**
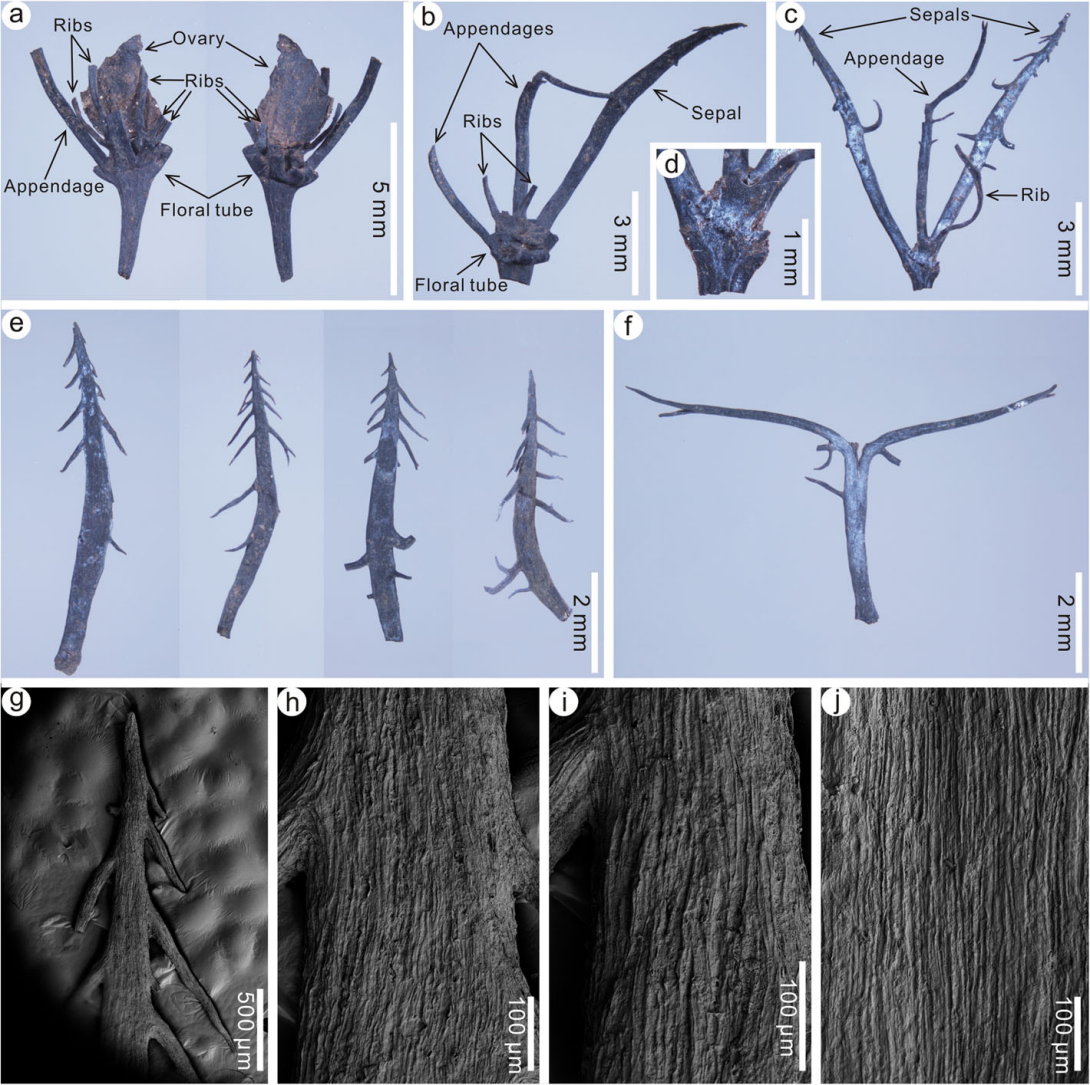
Floral cups and dispersed sepals and intersepal appendages of *Primotrapa weichangensis* from the Miocene of Weichang, China. **a** Front and back views of a floral cup showing the base of the fruit free from the shallow, saucer-shaped floral cup. PEPB70605. **b** A small saucer-shaped floral cup with remains of the sepals, epicalyx, and fruit ribs. PEPB70606. **c-d** Longitudinal view of a floral cup (**c**) and its enlargement (**d**). PEPB70607. **e** Four detached, isolated sepals showing sparsely spaced recurved barbs. PEPB70629–1–4. **f** A detached, isolated intersepal appendage. PEPB70630–1. **g-j** SEM of a sepal showing its striated surface. PEPB70629–5

**Fig. 5:**
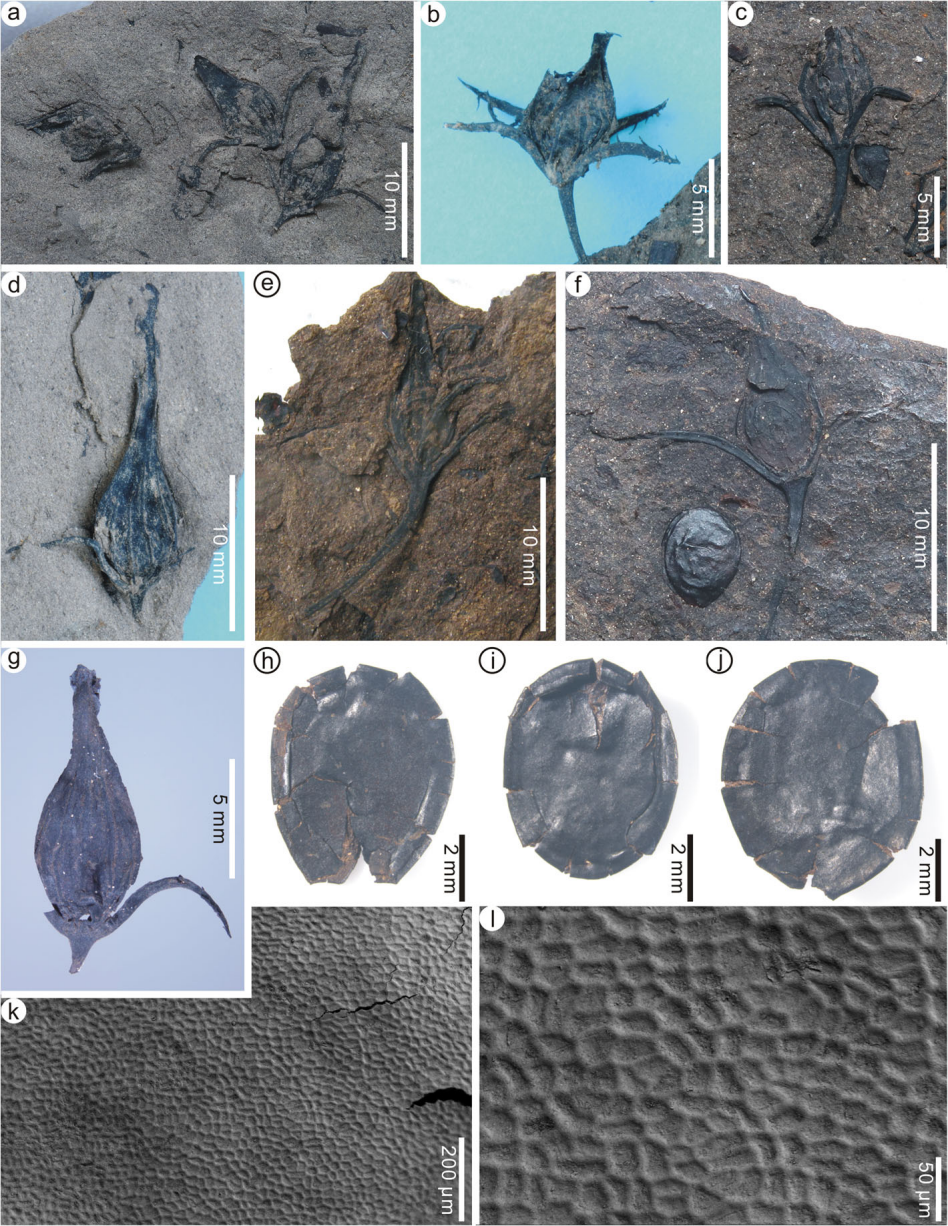
Fruits and seeds of *Primotrapa weichangensis* from the Miocene of Weichang, China. **a** Muddy siltstone with three *P. weichangensis* fruits exposed on the surface. PEPB70631. **b-g** Laterally compressed fruits, each showing a persistent floral cup with 1–4 remaining sepals, ribbed fruit surface, persistent peduncle, and probably one seed in the fruit (f). PEPB70632**–**70637. **h-j** Laterally compressed seeds of *P*. *weichangensis*. PEPB70681–70683. **k-l** SEM of the seed surface, showing irregularly shaped, pentagonal to hexagonal cells

*Paratypes*: PEPB70592–70593 (Fig. 3b, c), 70596–70598 (Fig. 3d–f), 70601–70607 (Fig. 3g–k, Fig. 4a–d), 70629–70630 (Fig. 4e, f), 70631–70637 (Fig. 5a–g), 70681–70683 (Fig. 5h–j).

*Etymology*: The species epithet refers to the fossil locality in Weichang County.

*Type locality*: Guangfayong Town, Weichang County, Hebei Province, China.

*Stratigraphy and geological age*: Hannuoba Formation, early Miocene.

*Repository*: National Museum of the Plant History of China, Chinese National Herbarium, Institute of Botany, Chinese Academy of Sciences, Beijing, China.

Description: The floral cups (hypanthia) of these fossil flowers are very short, shallow, saucer-shaped, perigynous, and usually persistent on the fruits (Fig. 3a–c, g–n); however, some hypanthia are found detached from the immature fruits (Fig. 3d–j). Sepals are four, acicular, curved outward at about their midpoint, 7–14 mm long and about 1 mm wide, and sparsely barbellate toward the apex (Figs. 3a–c, g–n, Fig. 4a–e), inserted on the floral axis in the same whorl and arising at a 30–40° angle. The barbs on the sepals are straight or recurved, forked once or unforked, about 1–2 mm long (Fig. 4e). The intersepal appendages are four, Y-shaped, spiny, dichotomized once, 3–5 mm long, up to 0.5 mm wide at their bases, nearly the same length as the apical forks (Fig. 3a, g–j, Fig. 4f), and alternate with the sepals at the rim of the floral cup. The epidermis of the sepals is composed of elongated cells (Fig. 4g–j). At the bottom of the floral cup is a single superior ovary (Fig. 3k–n). The pedicel is very long (Fig. 3a–c).

Fruits are small, 7.0–16.0 mm long, 3.0–4.5 mm wide, fusiform or ovoid with an elongated apex, obtuse base, sparsely ribbed surface (Fig. 5a–g). Pericarps are thinner and less woody than *Trapa*. The ribs are probably the remains of a fleshy exocarp, and tightly adnate to the endocarp, acicular, arising longitudinally from the base of the fruit, and extending beyond the broadest part of the fruit, but not reaching the apex of the fruit (Fig. 5d). Sepals are persistent, mostly not barbellate (Fig. 5a–g). Intersepal appendages are absent in mature fruits (Fig. 5a–g), probably due to abscission. Peduncles are slender, curved and broadening towards the floral cup, up to 4 cm long, 1–2 mm wide (Fig. 5a–g). Fruits are likely one-seeded (Fig. 5f) and irregularly dehiscent (Fig. 5b, c, f). Seeds are obovoid or elliptoid, 5.4–7.5 mm long and 4.9–6.5 mm wide (Fig. 5h–j). The surface of the testa consists of irregularly shaped, pentagonal to hexagonal cells (Fig. 5k, l).

### *Primotrapa pomelii* (Boulay) Y. Li et C.-S. Li comb. nov.

Synonyms:

1878 *Carpolithus pomelii* Saporta [31], p. 67, nom. inval.

1899 *Trapa pomelii* Boulay [32], p. 66, pl. 9, figs. 98-100

1991 *Hemitrapa pomelii* (Saporta) Mai [33], p. 102, pl. 13, figs. 6-10

2003 *Hemitrapa* cf. *pomelii* (Boulay) Mai [34], fig. 2

Occurrences: *P*. cf. *pomelii* was reported from the late Eocene diatomite of Kučlín, Czech Republic [34], while *P*. *pomelii* occurs in the late Oligocene of Rott, Germany, the early Miocene of Otterwisch, Germany, and the early Miocene of Gergovie, France [32–36].

Remarks: This species represents the oldest fossil species of the subfamily Trapoideae and is morphologically much different from the majority of the species of *Hemitrapa*. It is transferred here from *Hemitrapa* to *Primotrapa*, because its sepals are inserted near the base of the fruit [32–36] as they are in *Primotrapa*, rather than inserted about mid-length along the fruit as is characteristic for *Hemitrapa* [9, 17, 27]. The main difference between *P*. *pomelii* and *P*. *weichangensis* is that the fruit surface of *P*. *pomelii* is more ribbed [32, 34, 36] than *P*. *weichangensis* (Fig. 5a–g), and the fruit apex gradually narrows distally into a conical structure in *P*. *pomelii* [32, 34, 36], instead of the narrowly elongated apex seen in *P*. *weichangensis* (Fig. 5a–g). However, some specimens of *P*. *pomelii* from the late Oligocene of Rott and the early Miocene of Otterwisch, Germany [33, 35, 36] show definite similiarities to *P*. *weichangensis*, including some immature fruits with a shallow, saucer-shaped floral cup, as observed in our specimens (Fig. 3e–j). However, the floral cups of the German fossils are very incomplete and lack complete sepals and intersepals, hampering more detailed comparison. Furthermore, the barbs on their sepals are different, arising at a narrow angle to the sepal, as in *Trapa* (Fig. 1f), whereas in our species the barbs arise at a wide angle to the sepal (Fig. 4e).

### *Primotrapa alpina* (T. Su et Z.-K. Zhou) Y. Li et C.-S. Li comb. nov.

Synonym:

2018 *Hemitrapa alpina* T. Su et Z.-K. Zhou [37], pl. 2, figs. 1-10

Occurrence: Earliest Oligocene of Kajun, Markam County, Xizang, China.

Remarks: The fruits of *Primotrapa alpina* are elongate or fusiform in shape and are not borne on persistent peduncles, differentiating them from *P*. *pomelii* and *P. weichangensis*. However, the absence of a peduncle in *P. alpina* may be due to taphonomic bias or an abscission of a mature fruit from the peduncle at maturity [37]. Most specimens of *P. alpina* possess a basal, inferior ovary, whereas our specimens of *P. weichangensis* each bear a superior ovary. Moreover, the apex of the fruit is finely ribbed and conical in *P. alpina*, while it is not ribbed and narrowly elongated in *P. weichangensis*.

## Discussion

### Carpological comparisons with related fossil and extant aquatic taxa

**Fig. 6:**
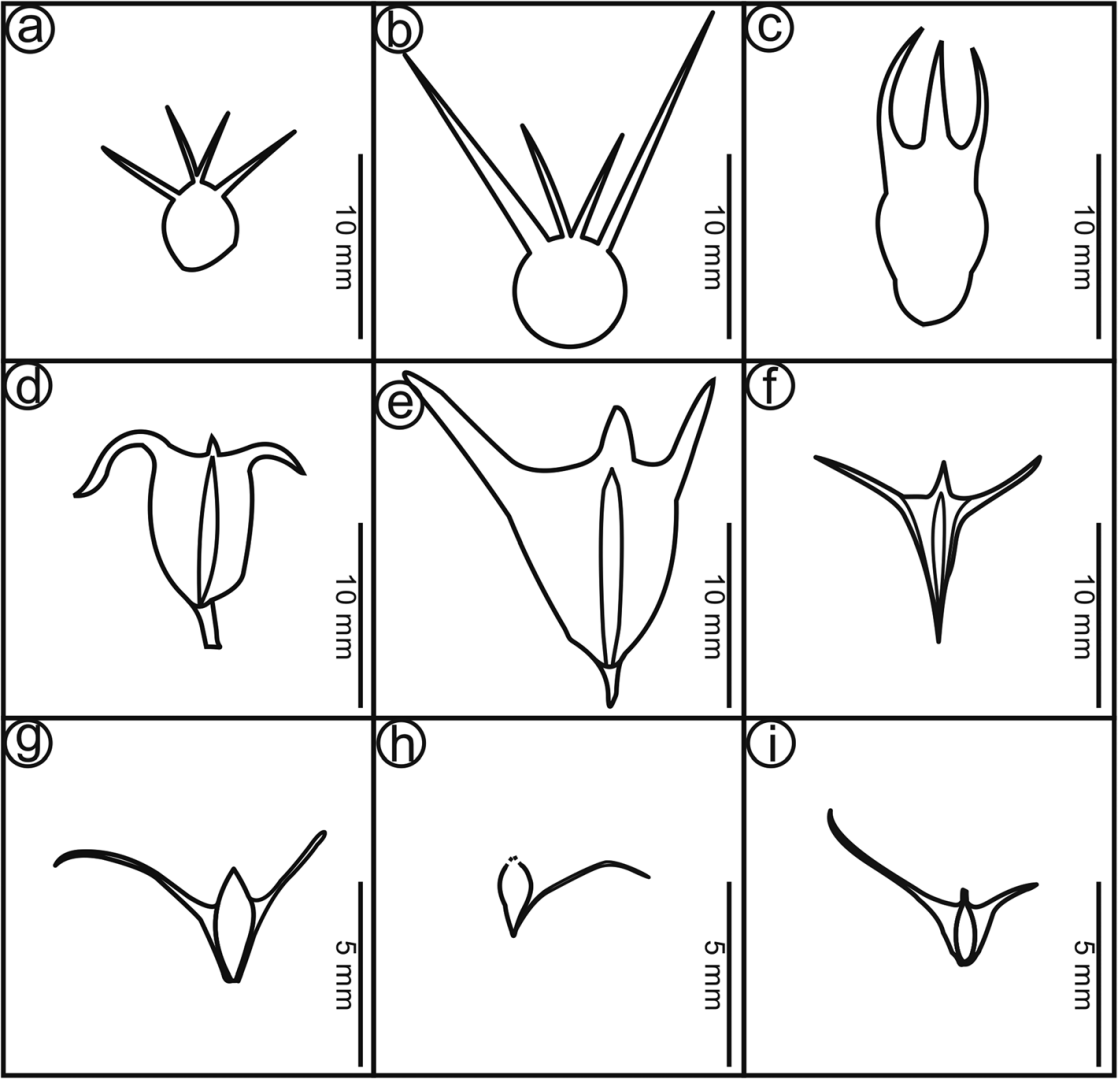
Line drawings of the fruits of the genera *Beipiaoa*, *Palaeotrapa* and *Prototrapa*. **a-c** *Beipiaoa parva* Dilcher, Sun et Zheng (**a**), *B. rotunda* Dilcher, Sun et Zheng (**b**), and *B. spinosa* Dilcher, Sun et Zheng (**c**) from the Early Cretaceous Yixian Formation of Beipiao, Liaoning, China [39]. **d-f** *Palaeotrapa aculeata* (Krysht.) Golovn. (**d**), *Palaeotrapa bicornata* Golovn. (**e**), and *Palaeotrapa triangulata* Golovn. (**f**) from the Late Cretaceous of the Koryak Upland, Russia [38]. **g-i** *Prototrapa douglasii* V. Vassiljev (**g**), *Prototrapa praepomelii* V. Vassiljev (**h**), and *Prototrapa tenuirostrata* V. Vassiljev (**i**) from the Early Cretaceous of southeastern Australia [40]

*Primotrapa* is superficially similar to three Cretaceous genera, i.e. *Beipiaoa* Dilcher, Sun et Zheng, *Palaeotrapa* Golovneva and *Prototrapa* Vassiljev, each of which consists of three species [38–40]. *Beipiaoa* is characterized by having apically three- or four-horned fruits, which were reported from the Early Cretaceous Yixian Formation of Beipiao, Liaoning, China [39, 41] (Fig. 6a–c). *Palaeotrapa* is represented by two-horned fruits found in association with *Quereuxia* leaves in the Maastrichtian of the Koryak Upland, Russia [38] (Fig. 6d–f). *Prototrapa* was established for very small fruits (only 1–3 mm long) with two long horns from the Aptian–Albian of southeastern Australia [40] (Fig. 6g–i). *Beipiaoa*, *Palaeotrapa*, and *Prototrapa* all have horned *Trapa*-like fruits with a poorly developed fruit head and a distinctive fruit body [27], but they lack barbellate sepals, intersepal appendages, and finely ribbed fruit surface, which differentiate all these three genera from *Primotrapa*. The fruit of *Trapa* is also quite dissimilar to these three genera [2, 27, 34].

**Fig. 7:**
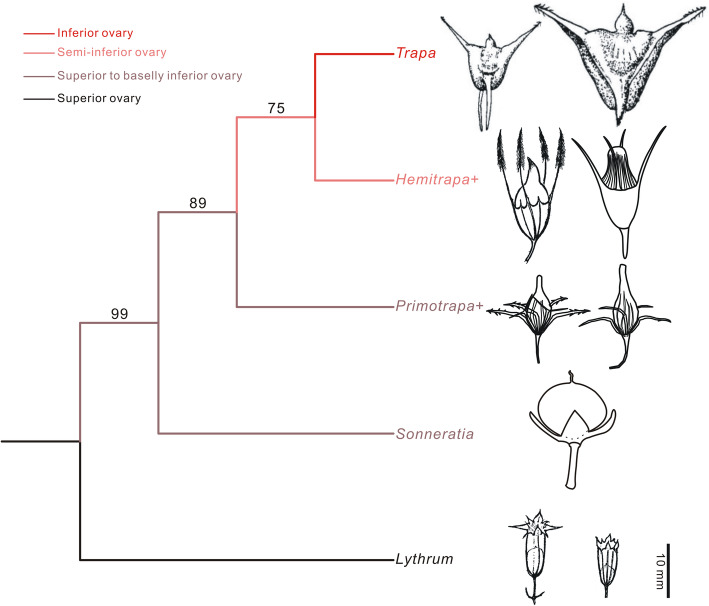
MP 50% Majority-rule consensus of 1416 trees and the line drawings of the fruits. Tree length = 19, Consistency Index (CI) = 0.947, Retention Index (RI) = 0.900, Rescaled Consistency Index (RC) = 0.853. Bootstrap values (50%) are above the branches. + indicates extinct genera. Scale bars = 10 mm for all fruits. The fruit of *Trapa* is represented by the extant species *T. incisa* Siebold et Zucc. on the left and *T. natans* L. on the right, both modified from Flora of China Editorial Committee [42]; *Hemitrapa* is represented by the type species *H*. *trapelloidea* Miki on the left, modified from Miki [9], and by the Chinese species *H*. *shanwangensis* Wang on the right, modified from Wang [27]; *Primotrapa weichangensis* is represented by an immature fruit on the left and a mature fruit on the right; *Sonneratia* is represented by the extant species *S. apetala* Buch.-Ham, drawn from Fig. 1a; *Lythrum* is represented by the fossil species *L*. *tetrasepalum* Miki on the left and the extant species *L. anceps* (Koehne) Makino on the right, both modified from Miki [9]

Instead, *Primotrapa* more closely resembles the now-extinct, Cenozoic genus *Hemitrapa*, as they both have fusiform fruits with a persistent floral cup, four distally barbellate sepals and four intersepal appendages arranged alternately at the rim of the floral cup, a less woody pericarp, a ribbed surface (*Primotrapa*) or a striated protrusion (the exserted portion) of the fruit (*Hemitrapa*), and a long, persistent peduncle. However, they differ in that *Primotrapa* has a very small, shallow, saucer-shaped floral cup, a superior to basally inferior ovary, and Y-shaped intersepal appendages, while *Hemitrapa* possesses a bowl-shaped floral cup, a semi-inferior ovary, and spine-like intersepal appendages (Fig. 7).

When compared with the fruits of extant aquatic plants, *Primotrapa* superficially resembles *Ceratophyllum* L. (Ceratophyllaceae) in having a similarly-sized, fusiform fruit with an elongated apex. However, *Ceratophyllum* differs in having two long basal spines and sometimes two more spines on the upper part of the fruit [43]. *Primotrapa* is similar to *Nymphoides* Ség. (Menyanthaceae) in having a perigynous floral cup deeply lobed to near its base, a superior ovary, and a long persistent peduncle. However, *Nymphoides* has (4- or) 5-merous flowers without intersepal appendages, and few-seeded capsular fruits [44].

*Primotrapa* is similar to *Trapa* in a series of characters, such as a persistent floral cup, four distally barbellate sepals and four intersepal appendages arranged alternately at the rim of the cup, and a ribbed fruit surface [42] (Figs. 1, 7). The main differences between *Primotrapa* and *Trapa* are that the former has a very small, shallow, saucer-shaped floral cup with a superior to basally inferior ovary, four sepals in the same level, Y-shaped intersepal appendages, and a long persistent peduncle, whereas the latter has a epigynous floral cup with a nearly completely inferior ovary, four sepals that initiate at the same level and later develop into 0 to 2 pairs of horns (sepals) at different levels, tubercle-like intersepal appendages (sometimes absent), and a sessile mature fruit with a distinctly contacted fruit neck (Figs. 1, 7). In summary, the fruit of *Primotrapa* is morphologically most similar to those of the extinct genus *Hemitrapa* and the living genus *Trapa*.

### Phylogeny and evolution of *Primotrapa* within Lythraceae s.l.

Our morphological phylogenetic analysis places *Primotrapa*, *Hemitrapa*, and *Trapa* in the monophyletic subfamily Trapoideae (Fig. 7). It also suggests that *Primotrapa* is the basal taxon of the subfamily, sister to a *Hemitrapa* + *Trapa* clade (Fig. 7). Derivation of *Sonneratia* and Trapoideae from a common ancestor is strongly supported by molecular evidence [10, 12–14] (Figs. 1, 7). As in *Primotrapa*, *Sonneratia* also has a superior to basally inferior ovary, which suggests that this type of ovary is a plesiomorphic character for *Sonneratia* and the Trapoideae.

*Lythrum*, an early-branching genus of Lythraceae s.l. (Fig. 1g), has 6-merous flowers [11]; however, a fossil *Lythrum* with 4-merous flowers was described from the late Miocene of Akazu and Obata, Japan, which was considered to represent a form ancestral to *Hemitrapa* [9]. In having a superior ovary, which is a plesiomorphic character of Lythraceae s.l. [10], *P*. *weichangensis* is potentially basal to *Primotrapa* and may also represent an example with primitive morphology of basal Lythraceae s.l. Interestingly, our investigation of the ontogeny of the fruit in extant *Trapa* (Fig. 1f) reveals that it has a nearly semi-inferior ovary in its early developmental stages, which gradually becomes nearly completely inferior as fruit matures. Thus, the ontogenetic development of *Trapa* fruit appears to reflect the phylogeny of the Trapoideae.

### Phytogeographic history of *Primotrapa*

**Fig. 8:**
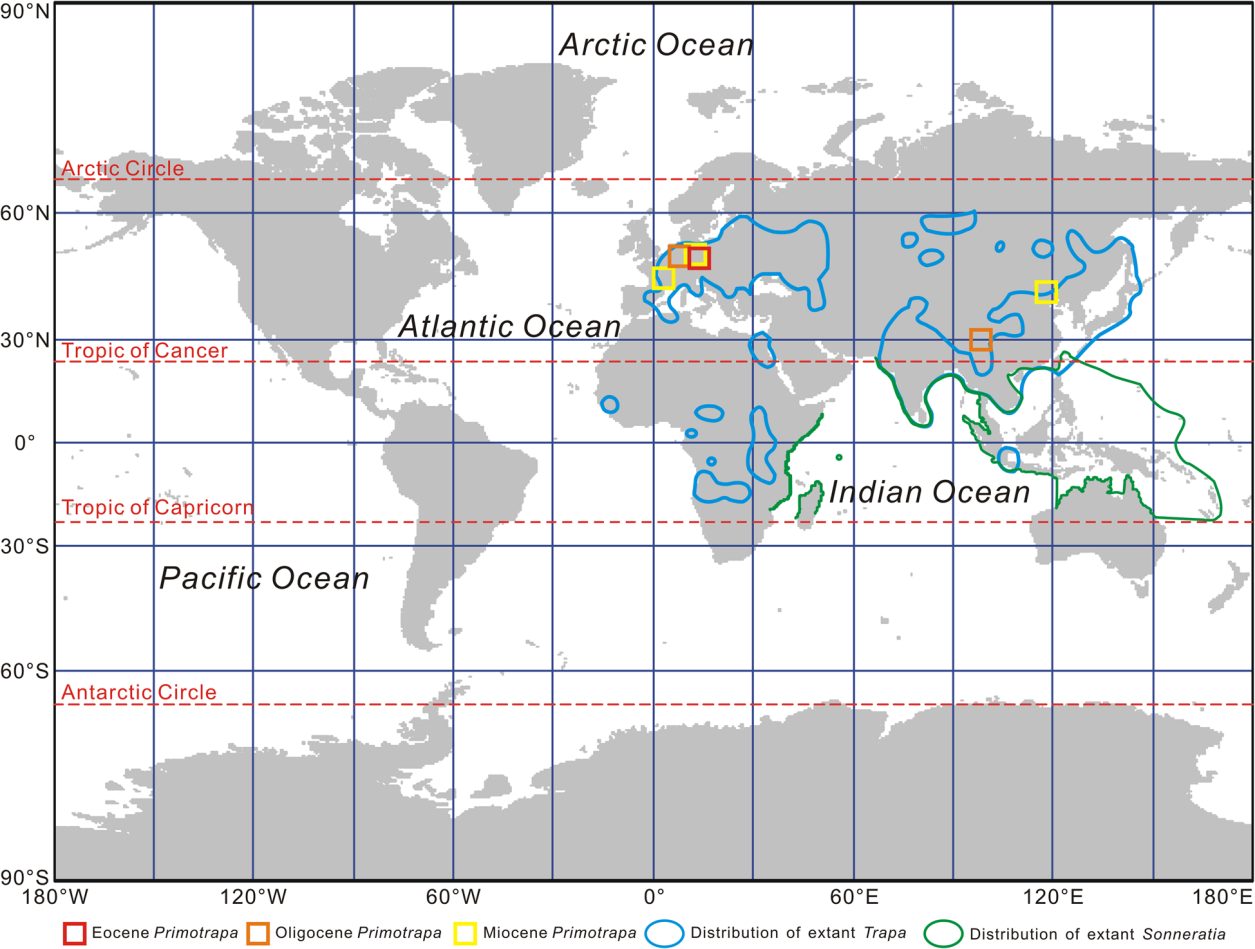
Distribution of fossil *Primotrapa*, as well as extant *Trapa* and *Sonneratia*, on a world map with Miller cylindrical projection. The base map is from https://www.naturalearthdata.com/. Fossil sites were plotted on the map using MapInfo Professional 8.5 SCP. The present-day distribution range of living *Trapa* and *Sonneratia* were modified from Mai [35] and Mao and Foong [45]

The oldest fossils of *Primotrapa* appear in the late Eocene diatomite of Kučlín, Czech Republic [34], then during the Oligocene, *Primotrapa* appears in Xizang, China [37], and Rott, Germany [33]. By the early Miocene, *Primotrapa* had spread to Otterwisch, Germany [33], Gergovie, France [32, 33], and Weichang, China (this paper). Overall, this pattern suggests that the genus likely originated during the Eocene of Eurasia, remaining restricted to the mid-latitudes of Europe and East Asia during the mid-Cenozoic (Fig. 8), then becoming extinct after the early Miocene. However, the last surviving member of subfamily Trapoideae, *Trapa*, has a broad current Old World distribution (Fig. 8). Similarly, its sister genus *Sonneratia* grows in Old World mangrove communities from East Africa to Indo-Malesia, Australia, New Guinea, and the West Pacific islands [11, 45] (Fig. 8).

## Conclusions

*Primotrapa weichangensis* Y. Li et C.-S. Li (Lythraceae s.l.: Trapoideae) is described as a new genus and species based on three-dimensionally preserved floral cups, fruits and seeds from the early Miocene of Weichang, Hebei Province, China. Two additional taxa are transferred to the new genus: *P. pomelii* (Boulay) Y. Li et C.-S. Li comb. nov. and *P. alpina* (T. Su et Z.-K. Zhou) Y. Li et C.-S. Li comb. nov. The close morphological similarity of *Primotrapa*, *Hemitrapa* and *Trapa* suggests a close evolutionary relationship within subfamily Trapoideae rather than convergent evolution, with morphological cladistic analysis placing *Primotrapa* as sister to the remainder of subfamily Trapoideae. In view of its superior ovary, a plesiomorphic character of Lythraceae s.l., *Primotrapa* seems to be intermediate between basal Lythraceae s.l. taxa (e.g. *Lythrum*) and more highly derived genera such as *Trapa*.

## Methods

### Geological setting

The fossil plant material described here was all collected from an outcrop at the Gaoshanjian locality (42°07′33″ N, 117°50′28″ E; 1406 ± 16 m), near Guangfayong Town, Weichang County, Chengde City, Hebei Province, China (Fig. 2). Collecting of fossils does not need any permits in this area. The outcrop consists of nine layers of lacustrine deposits (Fig. 2). The sedimentary rock and its underlying basalts belong to the Hannuoba Formation, according to the 1: 200,000 geological map of Qipanshan section (K-50-16) [29]. Fossil specimens of *Primotrapa* were collected from the third (muddy siltstone) and ninth layers (shale) (Fig. 2).

The age of Hannuoba Formation was originally assigned to the Miocene, based on the correlation with the presence of fossil mammals (*Monosaulax changpeiensis* Li and *Lagomorpha* gen. et sp. indet.) from Wafangyingzi, Zhangbei County, Hebei Province [46]. Later, it was re-interpreted as early Miocene based on plant fossils and sporopollen assemblages, as well as by K-Ar radiometric dating of the basalt (22.1 Ma) from Wuluogong, near Guangfayong Town [29]. Megafossils of mosses, e.g. *Leptodictyum*, *Drepanocladus*, and *Amblystegium* (Amblystegiaceae), conifers, e.g. *Pinus*, *Picea*, and *Tsuga* (Pinaceae), and angiosperms, e.g. *Comptonia* (Myricaceae), *Weigela* (Caprifoliaceae), *Scirpus* (Cyperaceae), and *Spirematospermum* (Zingiberaceae) have been described from the Miocene of Gaoshanjian [47–51]. Palynological investigation suggests a warm temperate mixed forest of conifers and broad-leaved trees with a few subtropical elements [30].

### Fossil preparation, photography and repository

The fossil remains of *Primotrapa* are charcoalified, and preserved as compressions in a matrix of muddy siltstone and lignite. They were exposed from the matrix by the technique of *dégagement* [52]. Selected specimens were treated with 10% HCl and 48% HF, and then rinsed with water and air dried. Specimens were imaged using a digital camera (Canon PowerShot G15), a stereomicroscope (ZEISS SteREO Discovery.V20), scanning electron microscope (LEO1530VP), and a Micro-CT (ZEISS Xradia520 Versa). In total, 40 floral cups, many detached sepals and intersepal appendages, 50 fruits, and 20 seeds were assigned the inventory numbers PEPB70591–70700 and deposited at the National Museum of the Plant History of China, Chinese National Herbarium, Institute of Botany, Chinese Academy of Sciences in Beijing. Ya Li and Cheng-Sen Li carried out the formal identification of the samples and provided details of the voucher specimens deposited.

### Morphological phylogenetic analysis

To reconstruct the phylogenetic relationship of *Primotrapa*, three extant genera and two fossil genera of Lythraceae s.l. were chosen for cladistic analysis, employing a morphological matrix of 15 characters (Tables 1 and 2). *Lythrum* was chosen as the outgroup because it is a basal member of the superclade I of the family (Fig. 1g), according to molecular phylogenetic results of Lythraceae s.l. [10]. Since the relationships among the extant genera could not be resolved by morphological data alone [10], the backbone constraint tree approach was used to determine the phylogenetic position of *Primotrapa*. The constrained tree is modified from the Bayesian tree of the combined molecular data proposed by Graham et al. [10]. All characters were unordered and equally weighted, except one character, namely, the position of the ovary. This character is considered to be irreversible, because a superior ovary is plesiomorphic in Lythraceae s.l., and semi-inferior to inferior ovaries are secondarily derived [10]. Maximum parsimony (MP) analysis was run in PAUP* 4.0a167 [53] using heuristic search algorithms with random addition (RA), 1000 replicates holding 1 tree at each step, tree bisection and reconnection (TBR) branch swapping and MULTREES settings, with the steepest descent option off. Bootstrap values were calculated from 10 replicates and RA sequences with 1000 replicates holding one tree.

**Table 1:** Character Coding for Morphological Analysis

Character	Codes
1. Merosity^a^	0 = 4; 1 = 6
2. Sepal barbs	0 = absent; 1 = present
3. Hypanthium shape	0 = cup-shaped; 1 = tube-shaped
4. Calyx lobe length^a^	0 = half or more of total floral cup/tube length; 1 = less than half of total floral cup/tube length
5. Hypanthium: carpel	0 = less than 1/2; 1 = ca. 1/2 or more
6. Pollen shape^a^	0 = prolate to prolate-spheroidal; 1 = oblate to oblate-spheroidal
7. Pollen pseudocolpi^a^	0 = absent; 1 = 3 pseudocolpi; 2 = 6 pseudocolpi
8. Pollen exine sculpture^a^	0 = psilate-scabrate to verrucate; 1 = striate
9. Ovary position	0 = superior; 1 = superior to basally inferior; 2 = semi-inferior; 3 = inferior
10. Fruit type^a^	0 = capsule; 1 = berry; 2 = drupaceous
11. Fruit surface ribs	0 = absent; 1 = present
12. Fruit neck	0 = absent; 1 = present
13. Seed number per fruit	0 = numerous; 1 = one-seeded
14. Seed compression^a^	0 = not compressed; 1 = slightly to strongly compressed
15. Pedicel	0 = persistent; 1 = detached

Note: ^a^indicates characters and codes cited from Graham et al. [10]

**Table 2:** Morphological Data Matrix

Taxon	1^a^	2	3	4^a^	5	6^a^	7^a^	8^a^	9	10^a^	11	12	13	14^a^	15
*Hemitrapa*	0	1	0	0	1	1	0	0	2	2	1	0	1	0	0
*Lythrum*	1	0	1	1	1	01	1	1	0	0	0	0	0	1	0
*Primotrapa*	0	1	0	0	0	?	?	?	1	2	1	0	1	0	0
*Sonneratia*	0	0	0	0	0	0	1	0	1	1	0	0	0	1	0
*Trapa*	0	1	0	0	1	1	0	0	3	2	1	1	1	0	1

Note: See Table 1 for codes. ? = missing data; ^a^indicates character matrix cited from Graham et al. [10]

## Data Availability

All data generated or analyzed during this study are included in this published article.

## Abbreviations

s.l.: *Sensu lato*
s.str.: *Sensu stricto*
TBR: Tree bisection and reconnection
RA: Random addition
CT: Computer tomography
